# Impact of intermittent preventive treatment with sulphadoxine-pyrimethamine targeting the transmission season on the incidence of clinical malaria in children in Mali

**DOI:** 10.1186/1475-2875-7-123

**Published:** 2008-07-08

**Authors:** Alassane Dicko, Issaka Sagara, Mahamadou S Sissoko, Ousmane Guindo, Abdoulbaki I Diallo, Mamady Kone, Ousmane B Toure, Massambou Sacko, Ogobara K Doumbo

**Affiliations:** 1 Malaria Research and Training Center, Departments of Epidemiology of Parasitic Diseases, Faculty of Medicine Pharmacy and Dentistry, University of Bamako, P.O. Box 1805, Bamako, Mali; 2 Department of Public Health, Faculty of Medicine Pharmacy and Dentistry, University of Bamako, P.O. Box 1805, Bamako, Mali

## Abstract

**Background:**

Recent studies have shown that intermittent preventive malaria treatment (IPT) in infants in areas of stable malaria transmission reduces malaria and severe anaemia incidence. However in most areas malaria morbidity and mortality remain high in older children.

**Methods:**

To evaluate the effect of seasonal IPT with sulphadoxine pyrimethamine (SP) on incidence of malaria disease in area of seasonal transmission, 262 children 6 months-10 years in Kambila, Mali were randomized to receive either IPT with SP twice at eight weeks interval or no IPT during the transmission season of 2002 and were followed up for 12 months. Subjects were also followed during the subsequent transmission season in 2003 to assess possible rebound effect. Clinical malaria cases were treated with SP and followed to assess the *in vivo *response during both periods.

**Results:**

The incidence rate of malaria disease per 1,000 person-months during the first 12 months was 3.2 episodes in the treatment group vs. 5.8 episodes in the control group with age-adjusted Protective Efficacy (PE) of 42.5%; [95% CI 28.6%–53.8%]. When the first 16 weeks of follow up is considered age-adjusted PE was 67.5% [95% CI 55.3% – 76.6%]. During the subsequent transmission season, the incidence of clinical malaria per 1000 persons-days was similar between the two groups (23.0 vs 21.5 episodes, age-adjusted IRR = 1.07 [95% CI, 0.90–1.27]). No significant difference was detected in *in vivo *response between the groups during both periods.

**Conclusion:**

Two malaria intermittent treatments targeting the peak transmission season reduced the annual incidence rate of clinical malaria by 42.5% in an area with intense seasonal transmission. This simple strategy is likely to be one of the most effectives in reducing malaria burden in such areas.

**Trial Registration:**

Clinicaltrials.gov NCT00623155

## Background

Malaria is one of the most common infectious diseases in the world. It is estimated that malaria causes between 300 and 500 million clinical cases and 700,000 to 1.6 million deaths each year with 94% of deaths occurring in sub-Saharan Africa [1,2].

In Mali, malaria is the leading cause of mortality and morbidity in the general population [3]. Like in many other Sahelian west African countries, malaria transmission is highly seasonal occurring during the rainy season, which varies from three to six months. It has been shown that more than 80% of malaria cases occurred during five months of the transmission season in the north savanna area of Mali. In such conditions, a suitable control strategy implemented during this period may have the most impact on the reduction of disease burden.

In the absence of vaccines, early case management and the use of insecticide-impregnated material are the best strategies to control malaria. However, their implementation has been slow and difficult to achieve due to the overarching poverty and illiteracy of the population as well as the insufficiency and inaccessibility of health services in most endemic areas. Home- and self-treatment have been proposed as alternatives to the insufficiency and inaccessibility of the health services, but these strategies have also several disadvantages, including misdiagnoses, lack of compliance with drug regimens, use of inappropriate medicine, which could contribute to development of drug resistance and the non-recognition of the severity of symptoms [4,5]. Consequently in sub-Saharan Africa, the morbidity and mortality of malaria are increasing [6]. There is an urgent need for an easy and simple malaria control strategy.

Recent, randomized controlled trials conducted in areas of perennial malaria transmission have shown that intermittent preventive treatment (IPT) given at the time of childhood vaccinations reduced the incidence of the first episode of malaria and severe anaemia by more than 50% during the first year of life, without there being any rebound in the subsequent year [7,8]. However, in countries such as Mali, where malaria is highly seasonal and prevalent in older children, IPT in infants (IPTi) may not be the optimum way in which to use antimalarial drugs to prevent malaria. An alternative approach is to give intermittent preventive treatment to children at risk only during the rainy season [9]. The primary aim of this study was to evaluate the impact of two seasonal IPT (sIPT) with sulphadoxine-pyrimethamine (SP) given at eight weeks interval on the incidence of malaria disease in children of six months to 10 years of age in an area of seasonal transmission, in Kambila, Mali. Secondary aims were to assess the impact of this strategy on the *in vivo *response of *Plasmodium falciparum *to SP and the potential rebound effect of this strategy on the subsequent transmission season after its cessation.

### Study design and methods

#### Study site

The study was conducted in Kambila, a rural village of about 1,500 inhabitants located about 25 km from Bamako, the capital city of Mali. *Plasmodium falciparum *is hyperendemic with parasitaemia prevalence rates ranging from 40–50% in the dry season (November – May) and 70–85% in the rainy season (June – October). The bed net coverage in the area at the time of the survey was less than 5%. The village was chosen to represent an area with four to five months of seasonal malaria transmission. SP was at the time, the second-line antimalarial drug in Mali, and was recommended for chloroquine failures. In 1995, a previous study found SP efficacy to be greater than 99% in the neighbouring area [10].

#### Study participants and design

The study was an open randomized controlled clinical trial. After a census of the village population, subjects in the target age group were screened for inclusion and exclusion criteria. Subjects who met inclusion and exclusion criteria were randomized either to receive two intermittent preventive treatments with standard recommended treatment doses of SP or no intermittent preventive treatment. Randomization codes were computer generated using simple randomization technique and treatment allocations were provided within sealed opaque envelopes. The first treatment was given at the beginning of the transmission season in July 2002 and the second treatment eight weeks later in August 2002. Inclusion criteria for the study included: 1) parental or other legal guardian consent; 2) age six months to 10 years; 3) having no chronic illness or symptomatic malaria; 4) agreeing to seek initial medical care for all medical illness in the study clinic during the entire study period; 5) having no plan to travel for a long time during the study period. Specific exclusion criteria included children with a history of allergy to sulpha drugs or SP.

##### Follow-up

Subjects from both groups were actively and passively followed during the study periods. The active follow-up was done weekly and consisted of: 1) questioning the subjects or parent for presence within the two past days of symptoms consistent with malaria including fever, headaches, body aches, malaise, diarrhoea, vomiting, and abdominal pain; 2) recording the axillary temperature; 3) examining conjunctivae and palms for pallor indicating a profound anaemia. The passive follow-up was done through continuous availability of study clinicians to evaluate any medical complaint at anytime during the entire study period. Any subject, who complains of symptom consistent with malaria, with an axillary temperature greater or equal to 37.5°C, or with profound anaemia or jaundice, was given a complete medical exam and laboratory assessment including malaria smear and haemoglobin or hematocrit measurement. The criteria for discontinuing further follow-up for a volunteer were: 1) consent withdrawal, 2) volunteer missed three consecutives follow-up visits, 3) assessment of the study physician as to whether being in the study was in the best interest of the volunteer.

##### Treatment

Subjects who were diagnosed with uncomplicated malaria were treated immediately with standard doses of SP, and those who were diagnosed with severe malaria were treated with quinine according to the guidelines of National Malaria Control Programme. They remained in the study so that multiple episodes of malaria disease could be detected over the course of the study period. *In vivo *response to SP was assessed using the WHO 2003 protocol [11] on uncomplicated malaria cases. Clinical assessments were made on days 1, 2, 3, 7, 14, 21 and 28 following treatment with SP, and blood smears were obtained (by finger prick) on days 3, 7, 14, 21 and 28 to determine the *in vivo *SP treatment failure. At the end of every clinic visit, subjects and their parent/legal guardian were encouraged to return immediately to the study clinic and/or their primary health care system should any other new symptoms appear.

Standard recommended treatment doses of SP (Fansidar^®^, F. Hoffman-La Roche Ltd, Basel, Switzerland) was given for IPT and for treatment of episodes of uncomplicated malaria (1/4 tablet per 5 kg wt for age ≤12 years). All subjects were observed for at least 60 minutes for vomiting. If vomiting occurred within 30 minutes, the full dose was repeated and if it occurred within 60 minutes, 1/2 of the dose was repeated. Cases of SP failure, and cases of malaria disease occurring within two weeks of a periodic treatment in the IPT group, were treated with chloroquine at 25 mg/kg in three days. Severe and complicated malaria cases were treated with quinine 8 mg/kg three times a day for five days. Serious adverse events were monitored during the duration of the study.

#### Laboratory methods

Haematocrit was determined using microhaematocrit reading device after centrifugation (IECMicro-MB centrifuge) and haemoglobin concentration was determined using a portable analyzer (Hemocue, Lake Forest, CA). Parasitaemia was assessed by counting the number of asexual *P. falciparum *parasites on Giemsa-stained thick blood films until 300 leukocytes were observed by microscopists unaware of the treatment group of the subject. Parasite densities were converted assuming 7,500 leukocytes/μl. Routine quality control was performed on 10% of the slides, with a second microscopist re-examining the blood films while blinded to the previously recorded result. Differences in parasitaemia of more than 10% were resolved by an expert microscopist.

#### Study endpoints

The primary end point of the study was the incidence rate of clinical malaria, defined as uncomplicated or severe malaria for the assessment of protective efficacy and the rebound effect. Uncomplicated malaria was defined as signs or symptoms consistent with malaria either leading to treatment-seeking behaviour or reported during weekly follow-up visits accompanied by any level of parasitaemia. These signs and symptoms included fever at the time of evaluation (axillary temperature ≥37.5°C by digital thermometer), profound anaemia (conjunctival or palmar pallor), jaundice, report of fever within the last two days, lassitude, headache, body aches, diarrhoea, or abdominal pain. *Severe malaria *was defined according to the most recent WHO protocols: severe anaemia (defined as haemoglobin <5 g/dL); parasitemia >10% or 500,000/mm^3^, prostration, respiratory distress, bleeding, recent repeated seizures, coma or obtundation, inability to drink, or persistent vomiting. The secondary endpoint was the non PCR corrected adequate clinical and parasitological response (ACPR) for the *in vivo *response of *P. falciparum *to SP. ACPR of *P. falciparum *to SP was defined according the WHO 2003 protocol [11] a protocol already available in 2002.

#### Sample size

The sample size was computed using the primary end point which was the incidence rate of malaria disease. Using normal approximation of square root transformation of Poisson formula, 96 persons-years of follow-up in each group were needed in order to detect a reduction of 40% in the incidence malaria disease, with 90% power and 95% confidence level, based on the estimated incidence of 1.2 episodes per person-year in the non-treatment group. To account for loss to follow up a total of 262 subjects (131 for each group) were enrolled. Using equivalency study of interventions method a total of 120 *in vivo *tests per arm will give more than 80% power for a maximum difference of 7% in ACPR between the two arms assuming ACPR of 95%.

#### Statistical analysis

Intention to treat analysis was used. The person-time method was used to calculate the incidence rate of malaria disease and to account for variable length of follow-up. The incidence rates were calculated as number of malaria episodes divided by the number of person days of follow-up at risk in each group. The incidence rates in the two groups were compared adjusted for age as continuous variable, using Poisson regression models in overall and for each age category (<5 years and > = 5 years). Subjects were not considered at risk for 28 days after a treatment for malaria disease. Protective efficacy of two intermittent treatments with SP was computed as 1 minus the incidence rate ratio of malaria disease over the first 52 weeks. This was also computed for the first 16 weeks to allow comparison with other studies. Proportions of subjects with ACPR using WHO latest protocol [11] in the two groups were compared during the first year and the subsequent transmission season (53^rd ^to 75^th ^week in December 2003) using Pearson chi square or Fisher exact tests as appropriate. Parasite densities were log transformed and geometric means parasite densities were compared using Student t test. Data were entered and verified using MS Access and then exported to Stata (StataCorp, College Station, Texas, US) for analysis.

#### Ethical considerations

The protocol was approved by the Ethical Committee of Faculty of Medicine, Pharmacy and Dentistry of the University of Bamako, Mali. Community permission and written individual consent from parent or legal guardian were obtained before inclusion in the study.

#### Role of the funding source

The study was funded by the UNDP/World Bank/WHO Special Programme for Research and Training in Tropical Diseases (TDR), re-entry grant ID AI0828. Additional funds came from the President Clinton donation to MRTC through NIAID/NIH. The two institutions had no role in the design conduct analysis and reporting of the study. The corresponding author had full access to all the data and had final responsibility for the decision to submit for publication.

## Results

### Characteristics of the study participants

A total of 262 subjects were enrolled in this study (131 in each arm). Baseline characteristics are summarized in Table 1. There was no significant difference between the two groups at baseline in term of age, gender and malaria parasite prevalence.

**Table 1:** Baseline characteristics of the study participants

	sIPT + (n = 131)	sIPT- (n = 131)	p
Mean age in years (SD)	5.6 (2.9)	5.1 (2.8)	n.s.
Sex (% of male)	50.4	58.0	n.s.
Parasite prevalence (%)	33.6	37.4	n.s.
Lost to follow up first year (%)			
Week 1 to 52	11.4	11.4	n.s.
Week 53 to 75	5.3	3.8	n.s.

SD = standard deviation

sIPT - = control group

sIPT + = treatment group

n.s. not significant

### Loss to follow-up

**Figure 1:**
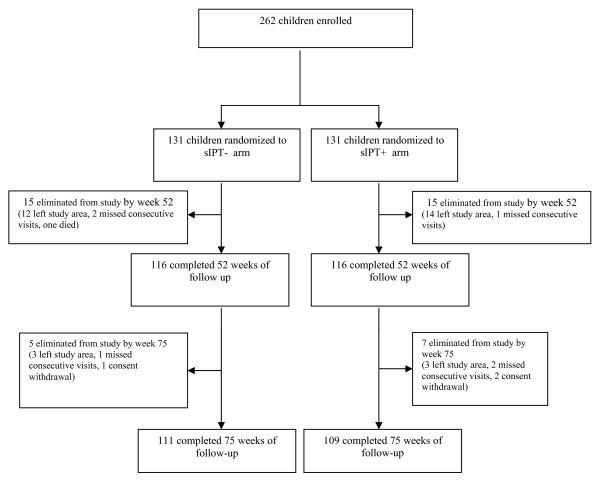
Flow Chart.

Of the 262 subjects enrolled, 30 subjects (11.4%) did not complete one year follow up and were equally distributed between the two groups (15 in each group). An additional 12 subjects (4.6%) did not complete the extended follow-up 7 in treatment group and 5 in the control group (Figure 1). Overall proportion of subjects who did not complete the follow up was similar between the two groups (16.8% versus 15.3%, p = 0.73). The reasons for loss to follow-up during the study were: migration to another location (n = 32), missing more three consecutive weekly visits (n = 6), consent withdrawal (n = 3) and death from severe malaria (n = 1).

### Incidence rate of malaria disease and efficacy of sIPT after 52 weeks of follow up

The incidence rate and protective efficacy after 52 weeks of follow up are presented in Table 2. Overall 352 episodes of malaria disease occurred, 221 in the control group and 130 in the sIPTc group. The incidence rates per 1000 person-days at risk for the malaria disease were 5.8 and 3.2 in the control and treatment groups respectively, giving a protective efficacy of 44.8% (95% CI 31.5% to 55.6%). After adjustment for age, the protective efficacy was 42.5% (95% CI 28.6 to 53.8%.) (Table 2). When the analysis was stratified by age categories, the incidence rate of malaria disease was 7.3 and 3.9 episodes per 1000 person-days in control and treatment groups respectively with an age-adjusted PE of 46.6% (95% CI 28.0% to 60.4%) in children less than five years of age. In children five years and above, the incidence rate of malaria disease were 4.5 and 2.7 episodes per 1000 person-days respectively in the control and treatment group. Age adjusted PE in this age group was 35.9% (95% CI 11.8% to 53.4%).

**Table 2:** Incidence rate of clinical malaria and protective efficacy after 52 weeks of follow-up per treatment group and age category

	<5 years		5–10 years		All	
			
	sIPT-	sIPT +	sIPT-	sIPT +	sIPT-	sIPT +
Number of malaria episodes	127	65	94	65	221	130
Person-days (-years) of follow up	17311 (47.6)	16590(45.6)	20799 (57.1)	24025 (66.0)	38110 (104.7)	40615 (111.6)
Incidence rate malaria disease per 1000 person-days (-years) of follow up	7.3 (2.67)	3.9(1.43)	4.5 (1.65)	2.7 (0.98)	5.8 (2.11)	3.2 (1.16)
Unadjusted PE in % (95% CI)	Reference group	46.6 (28.0–60.4	Reference group	40.1 (17.9–56.4)	Reference group	44.8 (31.5–55.6)
Age adjusted PE in % (95% CI)	Reference group	46.6 (28.0 – 60.4)	Reference group	35.9 (11.8–53.4)	Reference group	42.5 (28.6 – 53.8)

sIPT - = control group

sIPT + = treatment group

PE = Protective efficacy

Five cases of severe malaria (two cases of cerebral malaria, two cases of hyperparasitaemia, and one case of severe anaemia) occurred during the first 12 months of the follow-up, all in the control group giving an incidence rate of 0.048 episodes per 1000 persons-days at risk and a cumulative incidence of 3.9% in this group.

### Incidence rate of malaria disease and efficacy of sIPT after 16 weeks of follow-up

The incidence rate and protective efficacy after the first 16 weeks of follow-up are presented in Table 3. A total of 200 malaria disease episodes occurred during the first 16 weeks of follow-up, 145 in the control group and 55 in the group who received sIPT. The age adjusted PE in this period was 67.5% (95% CI 55.3% to76.6%) and was not significantly different in subjects of less than five years compared to those aged of five years and above.

**Table 3:** Incidence rate of malaria disease and protective efficacy after 16 weeks of follow up per treatment group and age category

	<5 years		5–10 years		All	
			
	sIPT-	sIPT +	sIPT-	sIPT +	sIPT-	sIPT +
Number of malaria episodes	86	30	59	25	145	55
Person-days of follow up	4797	5474	6207	7875	11004	13349
Incidence rate malaria disease per 1000 person-days of follow up	17.9	5.5	9.5	3.2	13.2	4.1
PE in % (95% CI)	Reference group	68.7 (57.4–77.1)	Reference group	66.6 (46.7–79.1)	Reference group	68.7 (57.4 – 76.1)
Age adjusted PE in % (95% CI)	Reference group	69.4 (53.6–79.8)	Reference group	63.4 (41.5–77.1)	Reference group	67.5 (55.3–76.6)

sIPT - = control group

sIPT + = treatment group

PE = Protective efficacy

### Incidence rate of malaria disease after cessation of sIPT during the next subsequent transmission season (weeks 53 to 75)

During the subsequent malaria transmission season in 2003, a total of 503 malaria episodes occurred, with 255 cases in the treatment group and 248 in the control group. The incidence rates of malaria disease were similar between the two groups 23.0 episodes per 1,000 persons-days in the treatment group and 21.5 episodes per 1,000 persons-days in the control group with age adjusted Incidence Rate Ration (IRR) = 1.07 (95% CI 0.90 to 1.27, p = 0.46). During this extended follow up period two cases of severe malaria occurred, both type cerebral malaria and in the control group.

### In vivo response of *P. falciparum *to SP

Of the 351 malaria episodes during the first year, valid *in vivo *tests were performed in 331 (94.3%) with 119/130 (91.5%) in the control group and 212/221 (95.9%) in the sIPT group. Table 4 shows that non PCR corrected 28 days Adequate Clinical and Parasitological Response (ACPR) was similar in the two groups in overall (92.9% in the control group vs. 94.1% in the sIPT group) and when the analysis was stratified by age categories. Mean parasitaemia at day 0 was also similar between the two groups with geometric means parasitaemia per μl of 11,480 (95% CI 8,770 to 15,027) in the control group versus 9,478 (95% CI 6,639 to 13,530) in the treatment group, p = 0.39.

**Table 4:** In vivo response of *P. falciparum *to SP during the first year of follow up group age categories and treatment groups

	< 5 years		5–10 years		All	
			
Outcome	sIPT- (n = 122)	sIPT + (n = 58)	sIPT- (n = 90)	sIPT + (n = 61)	sIPT- (n = 212)	sIPT + (n = 119)
ACPR	93.4	93.1	92.2	95.1	92.9	94.1
ETF	0.0	1.7	0.0	0	0.0	0.8
LCF	0.0	0.0	0.0	0	0.0	0.0
LPF	6.6	5.2	7.8	4.9	7.1	5.0

sIPT - = control group

sIPT + = treatment group

ACPR = adequate clinical and parasitological response

ETF = early treatment failure

LPF = late parasitological failure

During the extended surveillance following cessation of sIPT (53^rd ^to 75^th ^week), valid *in vivo *tests were performed in 472 (93.8%) of the 503 malaria episodes were treated with SP. Mean parasitaemia at day 0 was also similar between the two groups with geometric means parasitaemia per μl of 8,191 (95% CI of 6,367 to 10,538) in the control group versus 9,903 (95% CI 7,704 to 12,730) in the treatment group, p = 0.29.

*In vivo *responses per treatment and follow up period are presented in Table 5. The ACPR were similar between the two groups (87.9% versus 89.6%). The same similarities were found when the analysis was done in children less than five years of age or in those aged of five years and above.

**Table 5:** In vivo response of *P. falciparum *to SP during the extended period of follow up after cessation of sIPT implementation per age categories and treatment group

	1–4 years		5–11 years		All	
			
Outcome	sIPT- (n = 102)	sIPT + (n = 104)	sIPT- (n = 130)	sIPT + (n = 136)	sIPT- (n = 232)	sIPT + (n = 240)
ACPR	87.3	84.6	88.5	93.4	87.9	89.6
ETF	0.0	1.0	1.5	0.7	0.9	0.8
LCF	1.0	0.0	0.0	0.0	0.4	0.0
LPF	11.8	14.4	10.0	5.9	10.8	9.6

sIPT - = control group

sIPT + = treatment group

ACPR = adequate clinical and parasitological response

ETF = early treatment failure

LPF = late parasitological failure

Overall there was a reduction in the efficacy of SP during the extended follow-up compared to the first year of the follow up, especially in children less than five years of age.

#### Safety

No SP related serious adverse event was recorded during the study period. No subject was withdrawn because of allergy to SP.

## Discussion

This study assessed the impact of two doses of intermittent preventive treatment with SP at 8-week intervals in children of six months to 10 years of age targeting the transmission season in Kambila, Mali and found a reduction of 42.5% in annual incidence of clinical malaria. When SP plus single dose of artesunate was given at monthly intervals in an area with seasonal malaria transmission, Cisse *et al *[12] in Senegal found a reduction of 86% in incidence of clinical malaria children less than five years of age over 13 weeks period. This higher efficacy can be explained by the shorter time interval between treatments (four weeks instead of eight weeks), the number of intermittent treatments (three instead of two) and the shorter duration of the follow up (13 instead 52 weeks) [12]. Furthermore when the analysis of the efficacy of the two doses of intermittent preventive treatment with SP at 8-week intervals was limited to the first 16 weeks of follow up, the protective efficacy was 69.4% in children aged less than five years. Although the incidence rate of clinical malaria was higher in children less than five years of age compared to those of five years and above, the differences in efficacy of the strategy were not significantly different suggesting that IPT is also appropriate for older children.

While several studies have assessed the impact of IPT in pregnant women and infants, few studies have assessed the impact of this strategy in children. Several studies have shown that intermittent treatment given at 3, 6, 9 months of age reduced the incidence of clinical episodes by 20 to 60% with strong variations according to transmission duration and the seasonality [7,8,13,14]. In areas of seasonal malaria transmission, the burden of malaria remains high in older children in addition to infants [15-20]. Furthermore, more than 80% of the malaria cases in non-irrigated areas occurred during the 4–6 months of the transmission season [21], suggesting that sIPT is an appropriate preventive strategy for malaria control.

Unlike the use of other malaria control strategies such as use of insecticide-impregnated material that require daily implementation, this strategy requires only a twice-yearly treatment while offering significant protection against malaria. This simple strategy can be delivered through schools and/or local and national campaigns. Despite the current evidence and recommendation for use of insecticide-treated nets (ITN), this strategy remains largely under-utilized in Mali and many parts of Africa [22-24]. Increasingly, as efforts are being made to expand coverage of ITN it will be interesting to assess the efficacy of sIPT in children in the context of wide use of ITN.

The incidence rates of malaria disease (including severe malaria) reported in this randomized control trial were similar to those reported in previous studies in Mali [18,25]. This suggests an unbiased measure of the outcome despite the fact that the study was not a placebo controlled one, and that similar efficacy can be achieved by sIPTc in these areas.

There was no significant difference in SP efficacy *in vivo *between the treatment and control groups during the two periods of follow up using the WHO 2003 protocol for assessing the *in vivo *efficacy of antimalarials [11]. However there was a reduction in SP efficacy during the extended follow up period compared to the first year of follow up, especially among children less than five years of age. It is difficult to conclude if this reduction in SP in vivo efficacy was due to the sIPTc or was rather the expected decrease in efficacy due to the use of SP for treatment of malaria episodes in this population. In this study, uncomplicated malaria cases in both arms were treated with SP (2^nd ^line malaria treatment in Mali at the time of the study). While the higher efficacy and the longer prophylactic effect of SP compared to choloroquine (the first line treatment at the time of the study), provide more benefice for the study participants, this may contribute to the lack of difference in *in vivo *response between the two arms and the trend towards a lower efficacy during the extended follow up period.

Cisse *et al *[12] have found variation in frequency of molecular markers to SP resistance according to the season, without clear link with the intermittent preventive treatment. Results of studies undertaken by the IPTi consortium will bring more insight on the impact of IPT on drug resistance and whether this is an acceptable price to pay for the substantive benefit of this strategy and whether the combination with artesunate would be able to reduce the risk of increased resistance.

As shown in other studies [7,8,12] the cessation of the strategy was not associated with an increase of malaria disease during the subsequent transmission season. However in all these studies, IPT was given for a short time usually one year. It would be interesting to assess whether seasonal IPT in children given over longer periods impairs immunity to malaria.

In conclusion two malaria intermittent treatments targeting the peak of the transmission season have substantially reduced the incidence of clinical malaria in this area with intense seasonal transmission without a rebound effect after cessation. The strategy is simple and is likely to be very effective in reducing malaria burden in areas with seasonal malaria transmission.

## Completing interests

The authors declare that they have no competing interests.

## Authors' contributions

AD was the principal investigator of the study. He drafted the protocol, oversees the conduct of the study and contributed to the data analysis and interpretation. IS monitored the data quality of the study and contributed to the data analysis. MSS assisted with the implementation and coordination of the field activities. Data collection in the field data was done by OG, AID and MK. OBT assisted with the data management. MS contributed in overseeing the study. OKD contributed in the design of the study and in overseeing the data collection, analysis and interpretation. The manuscript was drafted by AD and all the authors contributed to revision and approved the final version.
